# Mortality and drug therapy in patients with chronic obstructive pulmonary disease: a network meta-analysis

**DOI:** 10.1186/s12890-015-0138-4

**Published:** 2015-11-11

**Authors:** David A Scott, Bethan Woods, Juliette C Thompson, James F Clark, Neil Hawkins, Mike Chambers, Bartolome R. Celli, Peter Calverley

**Affiliations:** 1 ICON Health Economics and Epidemiology, Seacourt Tower, West Way, Oxford, OX2 0JJ UK; 2 Centre for Health Economics, University of York, York, UK; 3 GlaxoSmithKline, Brentford, UK; 4 Brigham and Women’s Hospital, Harvard University, Boston, MA USA; 5 Institute of Aging and Chronic Disease, University of Liverpool, Liverpool, UK

**Keywords:** COPD, COPD treatment, Systematic review, Meta-analysis, Mortality

## Abstract

**Background:**

Increasing evidence suggests pharmacological treatments may impact on overall survival in Chronic Obstructive Pulmonary Disease (COPD) patients. Individual clinical trials are rarely powered to detect mortality differences between treatments and may not include all treatment options relevant to healthcare decision makers.

**Methods:**

A systematic review was conducted to identify RCTs of COPD treatments reporting mortality; evidence was synthesised using network meta-analysis (NMA). The analysis included 40 RCTs; a quantitative indirect comparison between 14 treatments using data from 55,220 patients was conducted.

**Results:**

The analysis reported two treatments reducing all-cause mortality; salmeterol/fluticasone propionate combination (SFC) was associated with a reduction in mortality versus placebo in the fixed effects (HR 0.79; 95 % Crl 0.67, 0.94) but not the random effects model (0.79; 0.56, 1.09). Indacaterol was associated with a reduction in mortality versus placebo in fixed (0.28; 0.08 to 0.85) and random effects (0.29; 0.08, 0.89) models. Mean estimates and credible intervals for hazard ratios for indacaterol versus placebo are based on a small number of events; estimates may change when the results of future studies are included. These results were maintained across a variety of assumptions and provide evidence that SFC and indacaterol may lead to improved survival in COPD patients.

**Conclusion:**

Results of an NMA of COPD treatments suggest that SFC and indacaterol may reduce mortality. Further research is warranted to strengthen this conclusion.

**Electronic supplementary material:**

The online version of this article (doi:10.1186/s12890-015-0138-4) contains supplementary material, which is available to authorized users.

## Background

Chronic obstructive pulmonary disease (COPD) is an important cause of morbidity across the world, and the third leading cause of death globally [1, 2]. Primary prevention by a combination of reducing tobacco exposure, decreasing contact with biomass fuels and noxious gases together with improved child health are the most effective ways of decreasing this burden in the longer term, although it takes time for the benefits of interventions on mortality to become apparent [3, 4]. In patients with symptomatic COPD the impact of specific medications on decreasing the risk of dying is an important consideration and merits scientific consideration. The evidence on mortality reduction from individual clinical trials in COPD is inconclusive with relatively few studies of duration and sample size sufficient to demonstrate an impact [5].

Network meta-analysis (NMA) provides a statistical approach to combining direct and indirect trial evidence to generate relative treatment effects between different drugs on outcomes of interest. In the absence of head-to-head trials including all comparators, NMA has been recommended by reimbursement agencies in the UK and Germany [6, 7] and endorsed by influential bodies such as ISPOR [8]. NMA has been applied to COPD mortality data on two previous occasions [9, 10].

We conducted a systematic review and network meta-analysis (NMA) designed to assess whether pharmacotherapy affects mortality reported in COPD clinical trials. NMA was then used to allow all treatment options to be compared in a single analysis [11–13]. The analysis combines survival data reported in two different forms: total number of deaths (r) from (n) subjects subsequently referred to as the ‘binary endpoint’, and hazard ratios which describe the impact of treatment on time to death and account for censoring. Although hazard ratios are more informative they are not reported in all studies and the inclusion of binary data enables the maximum number of trials to be included. Sensitivity analyses permitted us to analyse the robustness of the results to various assumptions supporting the base case analysis.

Primarily, our objective was to estimate the impact of specific COPD treatments on patient mortality using NMA. Secondly, we explored the strengths and limitations of undertaking and interpreting NMA in this context.

## Methods

### Systematic review

A systematic review was conducted to identify randomised, blinded trials of COPD patients treated with tiotropium, beclomethasone, budesonide, fluticasone propionate, triamcinolone, bambuterol, formoterol, salmeterol, salbutamol, indacaterol, theophylline, roflumilast, indacaterol maleate, ipratropium bromide, vilanterol trifenatate, fluticasone furoate or placebo. Dosing and administration method were not specified in the inclusion criteria. Combinations of the listed interventions were allowed; dose comparison studies were not included unless another listed intervention was also incorporated in the study. Studies were required to report all-cause mortality in binary or hazard ratio form for at least 24 weeks of follow-up; mortality could be reported as a study outcome or as a serious adverse event. Only English language full publications were included.

EMBASE (1988), MEDLINE, MEDLINE In-Progress (1946) and CENTRAL (1898) were searched from database inception to October 2012. Searches combined controlled-vocabulary and free-text terms for COPD and the treatments of interest; RCT filters were used in EMBASE and MEDLINE. Full publications were reviewed for inclusion by two analysts (JT and JC). Data was extracted from eligible trials by one analyst with validation conducted by the second analyst. Dosages of the same therapy were combined for the purposes of the analysis (indacaterol (150 μg od, 300 μg od, 600 μg od), budesonide (200 μg bid, 400 μg bid, 1200 μg od for 6 months followed by 800 μg od for 30 months), fluticasone propionate (250 μg bid, 500 μg bid), salmeterol (50 μg bid, 100 μg bid), formoterol (6 μg bid, 12 μg bid, 24 μg bid) and salmeterol fluticasone propionate combination (SFC) (50/ 250 μg bid, 50/ 500 μg bid)). The Cochrane risk of bias tool was used to assess methods of randomisation, allocation concealment, blinding, patient follow-up and incomplete reporting [14].

### Statistical analysis

Binary mortality data: total number of deaths (r) from (n) subjects, and hazard ratios with reported confidence intervals from published studies meeting our inclusion criteria were used as inputs to the analysis. We preferred hazard ratios over binary data where reported. Hazard ratios were taken from the Cox proportional-hazards models as these were consistently reported, in particular the Cox model for TORCH was preferred over that calculated directly from the Kaplan Meier in accordance with the other studies which reported HRs. Hazard ratio data and binary data were combined using the methodology established in Woods [15] which also appropriately incorporates multi-arm trials. Estimated treatment effects were synthesised using network meta-analysis (NMA) in a Bayesian multilevel framework. This method allows simultaneous comparison of outcomes of multiple treatments from trials comparing different sets of treatment options (providing a connected network of treatments can be formed) whilst retaining within-trial randomisation. A study protocol was written and reviewed prior to initiating the systematic review and analysis. Full details of the statistical method and the model code are provided in the Additional file 1.

The base case analysis included all RCTs meeting our inclusion criteria using the intention to treat (ITT) results from these studies, combining different licensed doses of the same medicines as single comparators. Results are presented for active treatments relative to placebo (reference).

### Sensitivity analyses

The following pre-planned analyses were conducted to examine the sensitivity of the study results to various assumptions:

(A) Including on-treatment (OT) mortality (excluding deaths that occurred to patients who ceased to receive the allocated study treatment) results in preference to ITT results where available.
(B) Meta-regression controlling for differences in COPD severity assuming a common covariable effect across treatments (assessed by baseline FEV_1_ % predicted – mean value per study)
(C) Excluding studies where patients had high lung function at baseline (mean FEV_1_ % predicted >65 %)
(D) Excluding studies where patients received unlicensed doses
(E) Excluding studies of less than 48 weeks duration
(F) Excluding studies not powered to detect a difference in mortality
(G) Excluding studies that failed to meet our specified quality assessment criteria (i.e. 2 or more components of the assessment had a high or unclear risk of bias) as assessed by the Cochrane Collaboration risk of bias [14]
(H) Including studies from the Dong [10] NMA for which mortality data were unavailable in the primary publication. Dong [10] cited a variety of sources for these data, including contacting the study authors and searching website and clinical trial registers; these were not included in the present base case analysis
(I) Separating patients treated with tiotropium by type of inhaler used (SoftMist or HandiHaler). Safety concerns (increased mortality risk) had at the time of the present analysis been raised around the SoftMist inhaler [16]. We also incorporated the results of TIOSPIR [17], a RCT of over 17,000 subjects designed to evaluate efficacy and safety of the two different inhalers, in this sensitivity analysis. TIOSPIR was not published until the final writing up of the present study.

Statistical models were fitted using WinBUGS [18]. As the present study is a Bayesian analysis we refer to credible intervals (the probability that the true value is contained within the interval) rather than confidence intervals; instead of statistically significant differences, we refer to important differences (95 % credible interval for hazard ratio does not cross 1.0).

Both fixed and random effect models were fitted. Fixed effect assumes there is one true effect of each treatment and that variation around this is attributed to chance whilst random effects assume a distribution of effects and that variance between studies is attributed to heterogeneity. Larger studies are thus attached relatively less weight in random effects model [19]. The Deviance Information Criteria (DIC) was calculated for each model and used to assess whether any model should be preferred [20]. Each model was run for a burn-in period of 40,000 simulations, which were then discarded, with parameter nodes monitored for a further 200,000 simulations. Caterpillar and Brooks - Gelman - Rubin (BGR) plots were used to compare results obtained using different initial values, thus ensuring that the models had converged [21].

## Results and discussion

### Systematic review

**Fig. 1:**
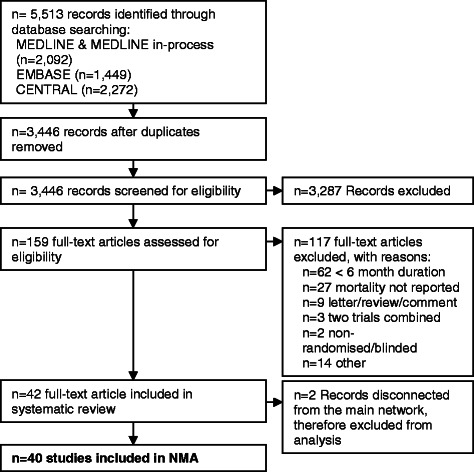
PRISMA diagram showing inclusion of studies at each stage of the systematic review and network meta-analysis

The systematic review identified 42 studies reporting all-cause mortality in COPD patients (Fig. 1; reasons for excluding full publications: Additional file 2: Table S1). Demographic characteristics of subjects (age, gender) are reported in Table 1; the impact of differences in baseline FEV_1_ % predicted is assessed in sensitivity analyses B and C. The proportion of current smokers was similar across trials, but three trials (all with patients with less impairment of lung function) reported levels in excess of 75 % [22–24].

**Table 1:** Baseline characteristics of included studies and all-cause mortality (binary data)

					Baseline characteristics					All-cause mortality (binary)		
References	Trial	Treatment	Dose	Study duration (weeks)	n	Mean age (yr.)	Women (%)	Current smokers (%)	FEV_1_ (mean % predicted)	Subjects	Deaths - ITT	Deaths – OT
[53]	Aaron 2007	Tiotropium + Salmeterol	18 μg od/50 μg bid	52	148	67.6	42.6	24.3	41.2	148	6	NR
		Tiotropium + SFC	18 μg od/50/500 μg bid		145	67.5	42.1	32.4	42.2	145	6	NR
		Tiotropium + Placebo	18 μg od		156	68.1	46.2	26.9	42.1	156	4	NR
[40]	Anzueto 2009	SFC	50/250 μg bid	52	394	65.4	49.0	42.0	41.2	394	4	NR
		Salmeterol	50 μg bid		403	65.3	43.0	43.0	40.0	403	6	NR
[54]	Bateman 2010	Tiotropium	5 μg od	48	1952	64.8	21.9	35.7	45.2	1952	52	NR
		Placebo	-		1965	64.8	23.0	35.9	45.4	1965	38	NR
[55]	ISOLDE (Burge 2000)	Fluticasone Propionate	500 μg bid	156	376	63.7	25.0	36.4	50.3	372	32	NR
		Placebo	-		375	63.8	26.0	39.2	50.0	370	36	NR
[56]	Calverley 2003	Budesonide + Formoterol	320/9 μg bid	52	254	64.0	22.0	33.0	36.0	254	5	NR
		Budesonide	400 μg bid		257	64.0	26.0	39.0	36.0	257	6	NR
		Formoterol	9 μg bid		255	63.0	25.0	36.0	36.0	255	13	NR
		Placebo	-		256	65.0	25.0	30.0	36.0	256	5	NR
[5]	TORCH* (Calverley 2007)	Fluticasone Propionate	500 μg bid	156	1534	65.0	25.0	43.0	44.1	1534	246	NR
		Salmeterol	50 μg bid		1521	65.1	24.0	43.0	43.6	1521	205	NR
		SFC	50/500 μg bid		1533	65.0	25.0	43.0	44.3	1533	193	NR
		Placebo	-		1524	65.0	24.0	43.0	44.1	1524	231	NR
[57]	Calverley 2007	Roflumilast	500 μg od	52	760	65.0	25.0	38.0	41.0	760	12	NR
		Placebo			753	64.0	24.0	35.0	41.0	753	20	NR
[58]	M2-124 (Calverley 2009)	Roflumilast	500 μg od	52	765	64.0	29.0	48.0	37.6	765	17	NR
		Placebo			758	63.0	29.0	48.0	37.5	758	17	NR
	M2-125 (Calverley 2009)	Roflumilast	500 μg od	52	772	64.0	21.0	35.0	34.6	772	25	NR
		Placebo			796	64.0	19.0	35.0	35.3	796	25	NR
[59]	Calverley 2010	Beclomethasone + Formoterol	200/12μg bid	48	232	63.0	20.7	38.8	41.9^a^	232	2	NR
		Budesonide + Formoterol	400/12μg bid		238	64.1	18.5	36.1	42.3^a^	238	4	NR
		Formoterol	12 μg bid		233	63.7	18.9	37.3	42.5^a^	233	0	NR
[49]	Campbell 2005	Formoterol bid + Formoterol	9 μg bid / 4.5 μg as needed	26	225	60.0	29.0	56.0	54.4^b^	225	1	NR
		Formoterol bid + Terbutaline	9μg bid / 0.5 mg as needed		215	60.0	39.0	54.0	53.0^b^	215	2	NR
		Placebo + Terbutaline	0.5 mg as needed		217	60.0	27.0	55.0	54.1^b^	217	0	NR
[26]	Casaburi 2005	Tiotropium	18 μg od	25	55	65.9	45.5	29.1	32.6^b^	55	1	NR
		Placebo	-		53	67.3	41.5	18.9	36.2^b^	53	0	NR
[27]	Chan 2007	Tiotropium	18 μg od	48	608	66.8	41.0	32.0	39.4^b^	608	15	13
		Placebo	-		305	66.9	39.0	30.0	39.3^b^	305	4	2
[60]	Choudhury 2007	Fluticasone Propionate	500 μg bid	52	128	67.6	52.0	40.6	53.2	128	3	NR
		Placebo	-		132	67.3	44.0	35.6	55.0	132	0	NR
[28]	INVOLVE (Dahl 2010)	Indacaterol (300)	300μg od	52	437	64.0	19.7	NR	51.5	437	1	1
		Indacaterol (600)	600μg od		425	63.0	23.1	NR	50.8	428	1	0
		Formoterol	12μg (bid)		434	64.0	19.8	NR	52.5	435	5	3
		Placebo	-		432	63.0	18.5	NR	52.0	432	5	4
[29]	Donohue 2002	Salmeterol	50 μg bid	26	213	64.6	25.0	NR	40.2^bc^	213	3	NR
		Tiotropium	18 μg od		209	64.5	26.0	NR	40.2^bc^	209	0	NR
		Placebo	-		201	65.6	25.0	NR	40.2^bc^	201	4	NR
[30]	INHANCE (Donohue 2010)	Indacaterol (150)	150 μg od	26	416	63.4	37.7	NR	56.1	416	1	NR
		Indacaterol (300)	300 μg od		416	63.3	36.8	NR	56.3	416	0	NR
		Tiotropium	18 μg od		415	64.0	35.2	NR	53.9	415	2	NR
		Placebo	-		418	63.6	39.0	NR	56.1	418	0	NR
[31]	Ferguson 2008	SFC	250/50 μg bid	52	394	64.9	42.0	40.0	39.8	394	6	NR
		Salmeterol	50 μg bid		388	65.0	48.0	38.0	40.6	388	3	NR
[32]	Hanania 2003	Fluticasone Propionate	250 μg bid	24	183	63.0	34.0	48.0	42.0^b^	183	0	NR
		Salmeterol	50 μg bid		177	64.0	42.0	51.0	42.0	177	0	NR
		SFC	250 / 50 μg bid		178	63.0	39.0	43.0	41.0	178	0	NR
		Placebo	-		185	65.0	32.0	47.0	42.0^b^	185	0	NR
[33]	VIVACE (Kardos 2007)	Salmeterol	50 μg bid	44	487	64.0	22.4	44.4	40.3	487	9	NR
		SFC	50/500 μg bid		507	63.8	26.0	40.6	40.4	507	7	NR
[41]	Kerstjens 1992	Ipratropium Bromide + Terbutaline	800/2000 μg bid	130	92	38.9	36.0	34.0	63.3^a^	92	0	NR
		Beclomethasone + Terbutaline	160/2000 μg bid		91	40.2	35.0	36.0	64.6^a^	91	0	NR
		Placebo + Terbutaline	na/2000 μg bid		91	39.6	36.0	37.0	63.3^a^	91	0	NR
[42]	INLIGHT-2 (Kornmann 2011)	Indacaterol	150 μg od	26	330	63.0	28.0	46.0	54.0	330	1	NR
		Salmeterol	50 μg bid		333	63.0	25.0	46.0	53.0	333	0	NR
		Placebo	-		335	64.0	23.0	45.0	53.0	335	3	NR
[34]	Mahler 2002	Fluticasone Propionate	500 μg bid	24	168	64.4	39.0	46.0	41.0^b^	168	0	NR
		Salmeterol	50 μg bid		160	63.5	36.0	46.0	40.0^b^	160	0	NR
		SFC	50 / 500 μg bid		165	61.9	38.0	46.0	41.0^b^	165	0	NR
		Placebo	-		181	64.0	25.0	54.0	41.0^b^	181	3	NR
[61]	Niewoehner 2005	Tiotropium	-	26	914	67.6	2.0	29.0	35.6^b^	914	22	NR
		Placebo	18 μg od		915	68.1	1.0	30.0	35.6^b^	915	19	NR
[22]	EUROSCOP (Pauwels 1999)	Budesonide	400μg bid	156	634	52.5	26.5	100.0	76.8^a^	634	8	NR
		Placebo	-		643	52.4	27.8	100.0	76.9^a^	643	10	NR
[35]	Rennard 2009	Budesonide + Formoterol	320/9 μg bid	52	494	63.2	37.7	39.1	38.6	494	8	3
		Budesonide + Formoterol	160/9 μg bid		494	63.6	37.2	41.9	39.6	494	8	6
		Formoterol	9 μg bid		495	62.9	34.7	45.1	39.3	495	6	2
		Placebo	-		481	62.9	34.7	43.9	40.8	481	8	4
[36]	FICOPD II (Rossi 2002)	Formoterol 12	12 μg bid	52	211	63.0	13.0	NR	47.0^b^	211	3	NR
		Formoterol 24	24 μg bid		214	62.0	17.0	NR	47.0^b^	214	1	NR
		Theophylline	200/300 mg bid		209	64.0	18.0	NR	46.0^b^	209	0	NR
		Placebo	-		220	63.0	21.0	NR	49.0^b^	220	0	NR
[62]	Schermer 2009	Fluticasone Propionate	500 μg bid	156	94	58.4	27.0	62.0	68.7^b^	94	8	NR
		Placebo	-		96	59.6	32.0	51.0	71.4^b^	96	3	NR
[23]	Shaker 2009	Budesonide	400 μg bid	208	127	63.6	38.0	100.0	51.0^b^	127	5	NR
		Placebo	-		127	63.6	46.0	100.0	53.0^b^	127	5	NR
[43]	Stockley 2006	Salmeterol	50 μg bid	52	318	62.3	24.0	46.0	45.8^b^	216	6	NR
		Placebo	-		316	62.4	23.0	47.0	46.1^b^	222	5	NR
[37]	Szafranski 2003	Budesonide + Formoterol	320/9 μg bid	52	208	64.0	24.0	30.0	36.0^b^	208	6	NR
		Budesonide	400 μg bid		198	64.0	20.0	36.0	37.0^b^	198	5	NR
		Formoterol	9 μg bid		201	63.0	24.0	38.0	36.0^b^	201	6	NR
		Placebo	-		205	65.0	17.0	34.0	36.0^b^	205	9	NR
[63]	Tashkin 2008	Budesonide + Formoterol	320/9 μg bid	26	277	63.1	32.1	44.4	39.1	277	3	NR
		Budesonide + Formoterol	160/9 μg bid		281	63.6	35.6	44.8	39.9	281	4	NR
		Budesonide + Formoterol	320 + 9 μg bid (separate)		287	63.7	25.8	41.5	39.2	287	0	NR
		Budesonide	320 μg bid		275	63.4	32.4	42.9	39.7	275	2	NR
		Formoterol	9 μg bid		284	63.5	34.5	41.9	39.6	284	1	NR
		Placebo	-		300	63.2	31.0	39.7	41.3	300	1	NR
[64, 65]	UPLIFT (Tashkin 2008/Celli 2009)	Tiotropium	18 μg od	208	2986	64.5	24.6	29.3	47.7	2987	446	381
		Placebo	-		3006	64.5	26.1	29.9	47.4	3006	495	411
[44]	Tonnel 2008	Tiotropium	18 μg od	39	266	64.9	13.2	23.7	47.5^b^	266	3	NR
		Placebo			288	63.5	14.6	30.2	46.2^b^	288	6	NR
[45]	COPE (van der Valk 2002)	Fluticasone Propionate	500 μg bid	26	123	64.1	14.6	22.0	57.5	123	1	NR
		Placebo	-		121	64.0	16.5	33.3	56.1	121	1	NR
[46]	CCLS (Vestbo 1999)	Budesonide	800 μg od + 400 μg od for 6 months; 400 μg bid for 30 month	156	145	59.0	41.4	75.9	86.2	145	4	NR
		Placebo	-		145	59.1	37.9	77.2	86.9	145	5	NR
[38]	Vogelmeier 2008	Formoterol	10 μg bid	24	210	61.8	24.3	NR	51.6^b^	210	0	NR
		Tiotropium	18 μg od		221	63.4	20.8	NR	51.6^b^	221	0	NR
		Tiotropium + Formoterol	18 μg od/10 μg bid		207	62.6	20.8	NR	50.4	207	0	NR
		Placebo	-		209	62.5	22.5	NR	51.1	209	1	NR
[47]	POET-COPD 2011 (Vogelmeier 2011)	Tiotropium	18 μg od	52	3707	62.9	25.6	48.0	49.2	3707	64	66
		Salmeterol	50 μg bid		3669	62.8	25.1	48.3	49.4	3669	78	73
[66]	INSPIRE (Wedzicha 2008)	SFC	50/500 μg bid	104	658	64.0	19.0	38.0	39.1	658	21	18
		Tiotropium	18 μg od		665	65.0	16.0	38.0	39.4	665	38	34
[24]	LHS (Wise 2000)	Triamcinolone	600 μg bid	156	559	56.2	36.0	90.5	68.5	559	15	NR
		Placebo	-		557	56.4	37.9	89.8	67.2	557	19	NR
[39]	Zheng 2007	SFC	50/500 μg bid	24	297	66.0	9.4	21.0	47.0^b^	297	2	NR
		Placebo	-		148	66.6	13.5	23.0	47.0^b^	148	0	NR
[48]	Zhong 2012	Budesonide + Formoterol	320/9 μg bid	26	156	65.7	1.9	NR	36.2	156	1	NR
		Budesonide	400 μg bid		152	64.7	7.9	NR	36.3	152	0	NR

FEV_1_ – mean % predicted, post-bronchodilator

*Powered to detect mortality

^a^Mean % predicted FEV_1_ is pre-bronchodilator

^b^Not stated whether mean % predicted FEV_1_ is pre-bronchodilator or post-bronchodilator

^c^FEV_1_ is mean of the three treatment arms

Assessment of study quality using the Cochrane risk of bias tool found that the quality of study reporting was generally high (Additional file 2: Table S2). Although all trials were randomised, 17 did not adequately describe the method of randomisation; [22, 24–39] and two studies did not adequately describe methods for allocation concealment [37, 39]. With the exception of FICOPD II where the theophylline arm (not included in analysis) was open-label [36], all studies were double-blind. Reporting of loss to follow-up was unclear in 17 studies; [22, 23, 27, 29, 30, 32, 34, 37, 40–47] imbalanced dropouts between the treatment groups in two studies was considered to result in a high risk of bias for the reported outcome data [39, 48]. In nine studies two or more components of the assessment were found to be potentially associated with an unclear or high risk of bias [22, 24, 27, 29, 30, 32, 34, 37, 39]. This was thought to reflect incomplete reporting rather than underlying methodological weakness in many cases.

### Studies included in the analysis

Two studies were excluded from the statistical analysis. Campbell [49], was excluded since the treatment arms in this trial (formoterol + formoterol as needed, formoterol + terbutaline as needed, placebo + terbutaline as needed) were not included in any of the other trials analysed, and therefore did not link to the evidence network. Similarly, Kerstjens [41], comparing terbutaline with ipratropium bromide + terbutaline and beclomethasone + terbutaline, did not connect to the main evidence network. Two treatments were excluded from the statistical analysis. Theophylline was included in a single trial, FICOPD II (Rossi [36]), which reported no deaths, and so it was not possible for a hazard ratio to be estimated for this treatment. Similarly, the only trial including tiotropium + formoterol combination (Vogelmeier [38]) did not report any deaths for this arm, which was therefore also excluded from the analysis. The other treatment arms of these studies were included in the analysis.

**Fig. 2:**
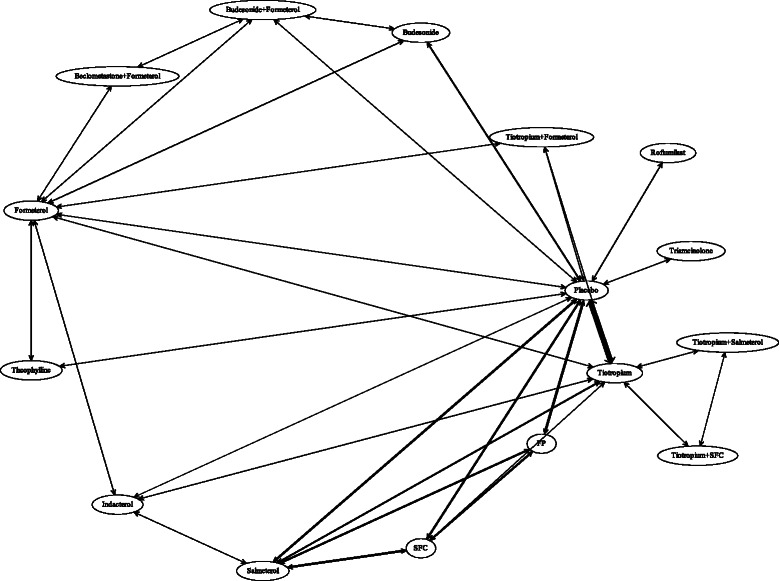
Base case evidence network. The width of the lines are proportional to the total person years of follow-up for all trials informing that comparison

The statistical analysis was based on 40 RCTs including 55,220 randomised subjects and 88,261 person years of experience, allowing the comparison of 14 treatments. Figure 2 shows the base case evidence network weighted by the number of person-years of follow up for each within-trial comparison. Reported binary mortality outcomes are presented in Table 1 and hazard ratios in Table 2. In the base case analysis hazard ratios for all-cause mortality were available for three studies and binary data were available for the remaining 37 studies.

**Table 2:** All-cause mortality (hazard ratios) of included studies

Trial	Treatment	Comparator	ITT				On treatment			
			HR	LCI	UCI	*p* value	HR	LCI	UCI	*p* value
TORCH	SFC	Placebo	0.811	0.670	0.982	0.030	NR	NR	NR	NR
	SFC	Salmeterol	0.946	0.777	1.151	0.580	NR	NR	NR	NR
	SFC	Fluticasone propionate	0.768	0.636	0.927	0.006	NR	NR	NR	NR
	Salmeterol	Placebo	0.857	0.710	1.035	0.110	NR	NR	NR	NR
	Fluticasone propionate	Placebo	1.056	0.883	1.264	0.550	NR	NR	NR	NR
UPLIFT	Tiotropium	Placebo	0.890	0.790	1.020	0.086	0.840	0.730	0.970	0.016
POET-COPD	Tiotropium	Salmeterol	0.810	0.580	1.130	0.210	0.850	0.610	1.190	0.350
INSPIRE	SFC	Tiotropium	NR	NR	NR	NR	0.480	0.270	0.850	0.012

### Base case results

**Fig. 3:**
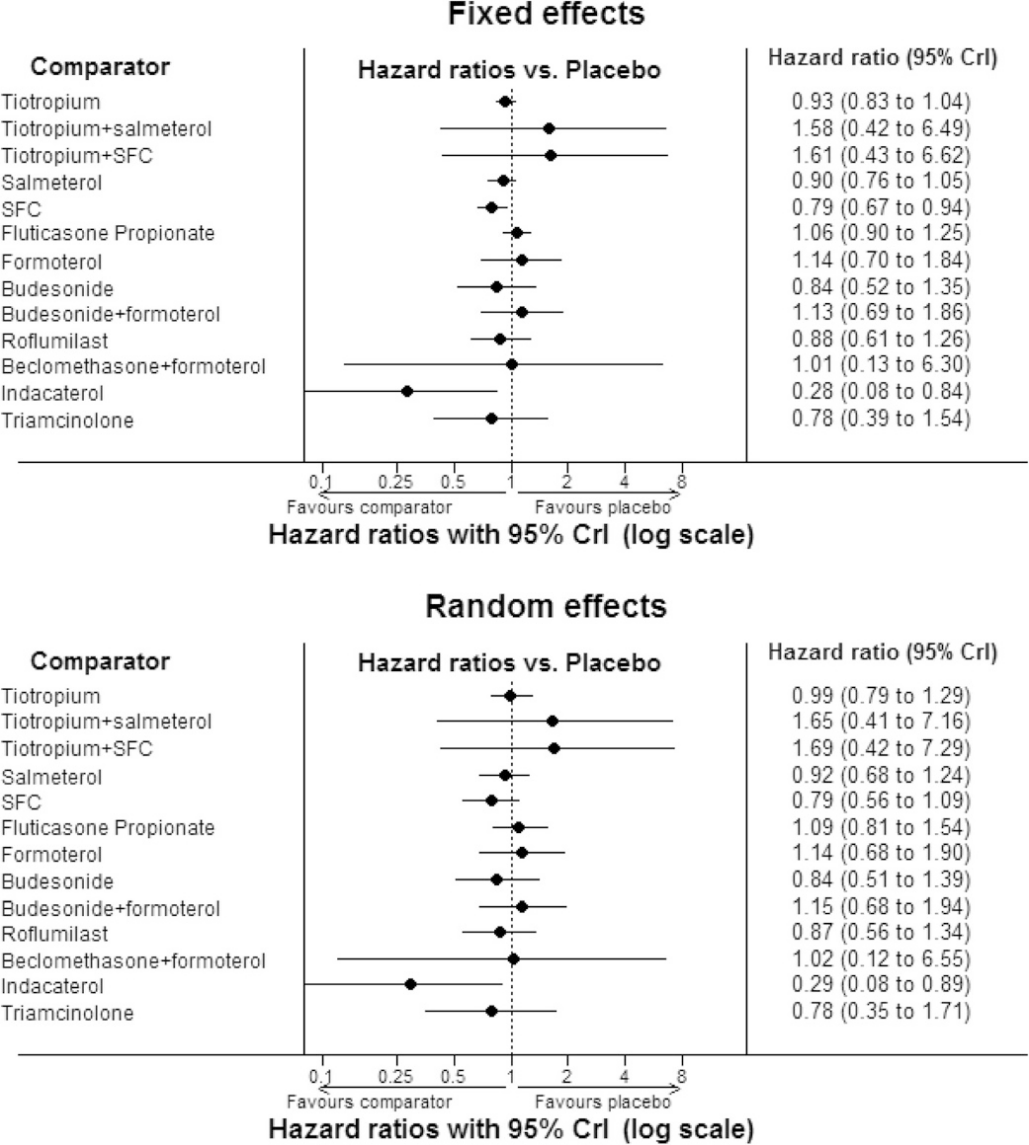
Forest plot of results of network meta-analysis. Hazard ratios compared to placebo (DIC 431.9 FE, 431.5 RE). SFC = Salmeterol fluticasone propionate combination; CrI = credible interval; Doses were pooled for the purpose of the analysis: indacaterol (150 μg od, 300 μg od), budesonide (200 μg bid, 400 μg bid, 1200 μg od for 6 months followed by 800 μg od for 30 months), fluticasone propionate (250 μg bid, 500 μg bid), salmeterol (50 μg bid, 100 μg bid), formoterol (6 μg bid, 12 μg bid, 24 μg bid) and salmeterol fluticasone propionate combination (SFC) (50/250 μg bid, 50/500 μg bid)

Results from the fixed and random effects base case analysis are presented in Fig. 3. Hazard ratios for each treatment are compared to placebo; a hazard ratio below 1.0 indicates that the treatment is associated with reduced mortality compared to placebo. There was no evidence to suggest that the random effects model was a better fit than the fixed effects model; a difference in DIC of 2–3 is required to be indicative of improved model fit [20]. However, if we believe there is true heterogeneity between the trials, the random effects model would be more appropriate.

Two interventions produced a hazard ratio relative to placebo that did not cross 1.0 using the fixed effects model. SFC was associated with a reduction in mortality of 21 % (HR 0.79; 95 % CrI 0.67, 0.94) and indacaterol with a mortality reduction of 72 % (HR 0.28; 95 % Crl 0.08, 0.85). Using a random effects model SFC failed to show evidence of effect (HR 0.79; 95 % CrI 0.56, 1.09). For indacaterol the result using the random effects model (HR 0.29; 95 % CrI 0.08, 0.89) was comparable to that using the fixed effects model. No evidence of effect on all-cause mortality (versus placebo) was found for other treatments. Although the results for most comparators have wide credible intervals suggesting inconclusive results, the HRs for tiotropium + salmeterol, tiotropium + SFC and beclomethasone + formoterol have particularly wide credible intervals; in each case the results are generated by single, relatively small study arms therefore the uncertainty around the estimates is high.

### Sensitivity analyses

Results of the sensitivity analyses did not in general differ markedly from the base case (Additional file 2: Table S3). For SFC vs placebo the relative treatment effect improved in the fixed effects analysis when unlicensed doses were excluded, but results from the random effects model showed no evidence of effect and were similar to the base case. Similarly, the relative treatment effect for indacaterol vs placebo strengthened slightly (HR 0.17, 95 % CrI 0.03, 0.78) when studies with a shorter duration were excluded.

## Conclusion

In this NMA, data from 40 trials were used to inform comparisons of mortality associated with 14 different pharmacological treatments for COPD. The method allows comparisons of treatments not compared directly within individual RCTs, and provides additional information on the relative efficacy of treatments for which direct trial comparisons are available. The results show that only indacaterol and the combination of the long-acting β_2_-agonist salmeterol and the inhaled corticosteroid fluticasone propionate (SFC) are associated with an important reduction in the risk of all-cause mortality in COPD in fixed effect models. Although the fixed effects model was presented as the base case there was no clear difference between the fixed and random effects models (both of which are presented). The results were consistent across a number of sensitivity analyses including controlling for disease severity.

Results for SFC are based on 233 deaths occurring in 7427 subject years. The results for indacaterol are based on four deaths occurring over 1446 subject years and have wide credible intervals. These results are sensitive to the number of deaths (a small change will have a large impact on the resulting HR) and may change with further research.

The results for many of the treatments are inconclusive, as demonstrated by the wide credible intervals exhibited around a number of the HRs. Whilst tighter credible intervals are observed around the results for tiotropium, salmeterol and fluticasone, our analysis is still inconclusive as to whether the treatments provide a greater benefit or harm to patients.

Two published NMAs have evaluated the relationship between pharmacological agents and mortality in COPD patients [9, 10]. Dong [10] considered all-cause mortality and cardiovascular death as outcomes: 42 trials published up to July 2011 were included, treatments were grouped by class (long-acting β2 agonists, inhaled corticosteroids etc.) and tiotropium was separated by inhaler type. The authors sourced trial mortality results from secondary sources. The study reported a reduction in mortality for LABAs combined with ICS compared with placebo (HR 0.80; 95 % CrI 0.67, 0.94) based on a fixed effects model. Baker [9] included 28 trials reporting the mortality published up to October 2007: treatments were grouped by class. A mortality reduction reported for LABAs in combination with ICS vs placebo (HR 0.71; 95 % CrI 0.49, 0.96) in the fixed effect model.

The present analysis included an additional 14 months of reported evidence and a wider range of treatments (roflumilast, indacaterol and triamcinolone) compared with Dong [10]. Furthermore, results were not aggregated by class. An assumption of class effects presupposes that the effect of each intervention within a class is identical. Even if the assumption holds for efficacy data it may not translate to safety data as interventions could have physiological effect other than the mechanism of action, therefore we chose estimate effects for each intervention independently [50].

Binary and hazard ratio data were combined in the same analysis, permitting the maximum number of studies to be included and using the best available data from each. We minimised the risk of errors by using data only from citable sources. Sensitivity analyses were undertaken to examine the robustness of the results to the underlying assumptions.

There are a number of limitations of this study. NMA methods depend on the assumptions that effect measures are additive on the selected scale and that relative treatment effects are comparable; [8] heterogeneity between trials may invalidate this assumption. Potential observed or unobserved differences between trials may impact on heterogeneity and thereby relative treatment effects.

The majority of the studies included were not specifically designed to capture mortality as a primary or secondary endpoint. The feasibility of conducting RCTs powered to detect differences in mortality in COPD patients is limited by the need for large sample sizes with sufficient follow-up, as well as the potential for introducing bias associated with differential dropout rates across study arms. Although this is a limitation of the current analysis, where there is an absence of head-to-head trials including all comparators, NMA is a useful tool for healthcare decision makers. In the present analysis we only included studies which reported mortality in the primary study publication. Inclusion of other studies where mortality is available in secondary publications may influence the results however the relatively small number of deaths in these trials makes this unlikely [10].

A potentially beneficial impact on mortality could be masked if a large number of studies with low or ineffectual dosages are included. Whilst there is some evidence that dose responsiveness may not be a significant factor in COPD [17, 51], this could be explored further by extending the network to incorporate dose finding studies and by implementing a three-level hierarchical NMA model with an additional level for each drug class [52].

Whilst we controlled for disease severity (recorded by baseline lung function) we did not control for other potential differences between trials which may impact on relative treatment effects (e.g. background therapy, history of exacerbations) as reporting was less consistent for these indicators.

Further work could examine baseline risk or the response in the placebo arms between studies. For example, similar rates of death per 1000 patient years (PY) were observed in the indacaterol (9.9/1000 PY), budesonide (10.0/1000 PY) and triamcinolone (11.4/1000 PY) placebo arms. Much higher rates were observed in the tiotropium (37.2/1000 PY), fluticasone propionate (43.3/1000 PY), salmeterol (47.0/1000 PY) and SFC (48.7/1000 PY) placebo arms (strongly influenced by the size and number of deaths in TORCH and UPLIFT) (Additional file 2: Table S4).

We conclude that currently available data from clinical trials in COPD suggest that some pharmacological treatments may have a significant impact on mortality, compared with placebo. In particular indacaterol and the combination of salmeterol and fluticasone propionate have shown evidence of reduction in all-cause mortality. The result for indacaterol is however based on a small number of deaths occuring to subjects receiving this therapy. Further research is warranted to strengthen our conclusions.

## Additional files

Additional file 1: Stastical Methods. (PDF 376 kb)
Additional file 2: Table S1. Reasons for excluding full publications. **Table 2.** Risk of bias results. **Table S3.** Fixed effects network meta-analysis results. Hazard ratios compared to placebo (95% Credible Intervals). **Table 4.** Placebo mortality by treatment arm. (PDF 352 kb)
