# Supplementary material for: Mortality and drug therapy in patients with chronic obstructive pulmonary disease: a network meta-analysis
Source: BMC Pulm Med. 2015 Nov 11;15:145. doi: 10.1186/s12890-015-0138-4 (PMC4642642; doi:10.1186/s12890-015-0138-4)
Supplement: Additional file 1: Stastical Methods. — (PDF 376 kb) [file 12890_2015_138_MOESM1_ESM.pdf]

Appendix

Statistical methods

The way in which the data are incorporated in to the model for a given study will depend on whether binary or hazard ratio data is reported, and for the hazard ratio data on whether the trial has 2 or more arms [1].

Hazard ratio data – two arm trials

The log hazard ratio statistics from two arm trials comparing treatments *k* to *b* is incorporated in the network meta-analysis model using a normal likelihood:

$$\bar{x}_{s,k,b} \sim \mathrm{N}\left(\ln\left(\frac{h_{s,k}}{h_{s,b}}\right), se^2_{s,k,b}\right) \qquad (1)$$

where $\bar{x}_{s,k,b}$ is the log hazard ratio estimate for study *s* comparing treatments *k* to *b* and $se^2_{s,k,b}$ is the corresponding variance.

The log hazard ratio estimates are then included in a treatment effect model with a linear regression structure, with the predicted log hazard ratio for a study s comparing treatments k and b equal to the difference between the two treatment coefficients:

$$\ln\left(\frac{h_{s,k}}{h_{s,b}}\right) = \beta_k - \beta_b \qquad (2)$$

where $\beta_1 = 0$ for placebo (the reference treatment) and β_b represents the treatment effect for the baseline treatment in study *s*. The β_k coefficient is equal to the log hazard ratio for treatment k compared to placebo.

Hazard ratio data – multi-arm trials

Estimates of relative treatment effects from trials with more than two treatment arms (“multi-arm trials”) will be correlated [2]. For example, hazard ratios comparing two different active treatments to placebo will be correlated due to their joint dependence on the time to event data in the placebo arm.

If a network meta-analysis is based on estimates of treatment effect in individual trial arms rather than estimates of relative treatment effect between arms (“contrast” statistics), this correlation will automatically be captured in the analysis [2]. This is the case for the studies reporting only binary data. However, when the network meta-analysis is conducted based on hazard ratio data, this correlation between arms will not automatically be captured [2].

For multi-arm trials reporting hazard ratio statistics, this problem can be addressed by converting the log hazard ratios (contrast statistics) to log hazards (arm-specific statistics). Log hazards for individual trial arms are derived by nominally setting the log hazard for the baseline treatment *b* for the trial to zero. The mean log hazards for the other treatments are then equal to the log hazard ratios compared to baseline treatment.

The variance for a log hazard ratio is the sum of the variances for the individual log hazards. Standard errors of the log hazards for each trial arm can therefore be estimated by solving

simultaneous equations based on the standard errors for the set of log-hazard ratios. For example:

$$se_b = \sqrt{\left((se^2_{k_1,b} + se^2_{k_2,b} - se^2_{k_1,k_2})/2\right)} \quad (3)$$

Where $se^2_{i,j}$ is the variance of the log hazard ratio comparing arm *i* to arm *j* and se_i is the standard error of the log hazard for arm *i*.

The standard errors of the log hazards for the other treatment arms are then estimated as:

$$se_k = \sqrt{se^2_{k,b} - se^2_b} \quad (4)$$

The log hazard statistics from multi-arm trials are incorporated in the analysis using the following likelihood functions. For the baseline treatment, *b*:

$$\bar{x}_{s,b} \sim \mathrm{N}\left(0, se^2_{s,b}\right) \quad (5)$$

For the other treatments:

$$\bar{x}_{s,k} \sim \mathrm{N}\left(\ln\left(h_{s,k}\right), se^2_{s,k}\right) \quad (6)$$

where $\bar{x}_{s,k}$ is the log hazard for treatment arm *k* from study *s* and $se^2_{s,k}$ is the associated variance.

The log hazard estimates are then included in a treatment effect model with a linear regression structure. The log hazard is estimated as the sum of a study specific ‘baseline’ term α_s and a treatment effect coefficient β_k :

$$\ln\left(h_{s,k}\right) = \alpha_s + \beta_k - \beta_b \quad (7)$$

where β_1 = 0 for placebo and β_b represents the treatment effect for the baseline treatment in study s. The fixed study level ‘baseline’ term is a nuisance parameter, included to ensure that the treatment effect estimates are informed by within trial differences between treatment arms and not by differences in baseline event rates across trials. The β_k coefficient is equal to the log hazard ratio for treatment k compared to placebo.

Binary data

The count data are incorporated in the network meta-analysis model using a binomial likelihood:

$$r_{s,k} \sim Bin(F_{s,k}, n_{s,k}) \quad (8)$$

where $r_{s,k}$ is the cumulative count of subjects who have experienced an event in arm *k* of study *s*; $n_{s,k}$ is the total number of subjects in arm *k* of study *s*; and $F_{s,k}$ is the cumulative probability of a subject having experienced an event (or ‘failure’).

A log cumulative hazard for each trial arm $\ln\left(H_{s,k}\right)$ is then derived from $F_{s,k}$.

$$\ln\left(H_{s,k}\right) = \ln\left(-\ln(1 - F_{s,k})\right) \quad (9)$$

The log cumulative hazard estimates are then included in a treatment effect model with a linear regression structure. The log cumulative hazard is estimated as the sum of a study specific 'baseline' term α_s and a treatment effect coefficient β_k:

$$\ln(H_{s,k}) = \alpha_s + \beta_k - \beta_b \tag{10}$$

where β_1 = 0 for the reference treatment (placebo in our example) and β_b represents the treatment effect for the baseline treatment in study *s*. The fixed study level 'baseline' term is a nuisance parameter, included to ensure that the treatment effect estimates are informed by within trial differences between treatment arms and not by differences in baseline event rates across trials.

Under an assumption of proportional hazards, the β_k coefficient is equal to both the log cumulative hazard ratio and the log hazard ratio:

$$\ln\left(\frac{\exp(\beta_k).h_{s,b}}{h_{s,b}}\right) = \ln\left(\frac{\int_0^t \exp(\beta_k).h_{s,b}}{\int_o^t h_{s,b}}\right) = \beta_k \tag{11}$$

where $h_{s,b}$ represents the hazard for the baseline treatment in study *s*. This identity allows us to combine the count statistics analysed on the log cumulative hazard scale with the hazard ratio data analysed on the log hazard scale.

As the β_k coefficient is equal to the log hazard ratio for the cumulative count data, the log hazard ratio data and the log hazard data, they can be combined within a single analysis.

Random effects

The treatment effect is modelled as both fixed and random effects. In a random effects analysis of a network containing multi-arm trial contrast data, the correlation in the random effects must also be taken in to account. Again this is due to the joint dependence of the multiple contrast estimates on common trial arms.

This correlation can be reflected in the model by separating the random effect deviation for each contrast in to the contributions to the random effect deviation of the two treatments that form the contrast. This is achieved by modifying the linear predictor component of the model for the cumulative count, log hazard ratio and log hazard data:

$$\ln(H_{s,k}) = \alpha_s + \beta_k - \beta_b + re_{s,k} - re_{s,b} \tag{12}$$

$$\ln\left(\frac{h_{s,k}}{h_{s,b}}\right) = \beta_k - \beta_b + re_{s,k} - re_{s,b} \tag{13}$$

$$\ln(h_{s,k}) = \alpha_s + \beta_k - \beta_b + re_{s,k} - re_{s,b} \tag{14}$$

where $re_{s,k}$ is the random effect deviation for arm k of study s and is assumed to be normally distributed with zero mean and variance $\sigma^2/2$ where σ^2 is the random effect variance for a treatment contrast:

$$re_{s,k} \sim N(0, \sigma^2/2) \tag{15}$$

This approach assumes that σ^2 is the same for all treatments and consequently that the random effect variance will be the same for all treatment contrasts. The assumption of a common random effect variance across treatment contrasts implies that the covariance for any pair of treatment contrasts from the same study will equal half the treatment contrast random effect variance [2].

A vague prior for the study specific baseline $\alpha_s \sim N(0,10^6)$ is used to ensure estimates of treatment effect are informed by within trial differences between treatment arms, and not by differences in absolute response between trials. A vague prior is also used for the treatment effect coefficients with $\beta_k \sim N(0,10^6)$ and $\beta_1 = 0$ (representing placebo).

The analysis will be conducted using WinBUGS [3], the code for which presented below. Bayesian software is typically used to conduct network meta-analyses [4,5].

Both fixed and random treatment effects model will be fitted as part of the analysis. In the random effects analysis the variance of the treatment effect is assumed to be the same for all treatments as there are insufficient data to estimate treatment-specific variances.

The Deviance Information Criteria (DIC) approach is used to compare the fit of the fixed and random effect models [6] and to select the preferred model. Caterpillar and Brooks, Gelman, Rubin (BGR) plots [7] are used to compare results obtained using different initial values, and thus ensure that the models have converged.

References

[1] Woods B, Hawkins N, Scott DA. Network meta-analysis on the log-hazard scale, combining count and hazard ratio statistics accounting for multi-arm trials: A tutorial. BMC Medical Research Methodology 2010; 10:54

[2] Salanti G, Higgins JP, Ades AE, Ioannidis JP. Evaluation of networks of randomised trials. Statistical Methods in Medical Research 2008. 17(3): 279-301.

[3] Spiegelhalter DJ, Thomas A, Best N, Lunn D. WinBUGS User Manual: Version 1.4. Cambridge: MRC Biostatistics Unit, 2003.

[4] Ades AE. A chain of evidence with mixed comparisons: models for multi-parameter synthesis and consistency of evidence. Statistics in Medicine 2003; 22(19):2995-3016.

[5] Lu G, Ades AE. Combination of direct and indirect evidence in mixed treatment comparisons. Statistics in Medicine 2004; 23(20):3105-24.

[6] Spiegelhalter DJ, Best NJ, Carlin BP, Van der Linde A. Bayesian measures of model complexity and fit (with discussion). Journal of the Royal Statistical Society 2002; 64:1-34.

[7] Brooks SP, Gelman A. General methods for monitoring convergence of iterative simulations. J Comput Graph Stat 1998;7:434–55.

WinBUGS code for fixed and random effects

Fixed effects model

```
model{

#Define Prior Distributions
	#On tx effect mean
	beta[1] <-0
	for (tt in 2:nTx){
	beta[tt]~dnorm(0,1.0E-6)
	}

	#On individual study baseline effect
	for(ss in 1:nStudies){
	alpha[ss] ~ dnorm(0,1.0E-6)
	}

#Fit data
	#For hazard ratio reporting studies
	for(ii in 1:LnObs ){
	Lmu[ii] <- alpha[Lstudy[ii]]*multi[ii] +  beta[Ltx[ii]] -
	beta[Lbase[ii]]
	Lprec[ii] <- 1/pow(Lse[ii],2)
	Lmean[ii] ~ dnorm(Lmu[ii],Lprec[ii])
	}

	#For binary data reporting studies
	for(ss in 1:BnObs){
	logCumHaz[ss] <- alpha[Bstudy[ss]] + beta[Btx[ss]] - beta[Bbase[ss]]
	cumFail[ss] <- 1-exp(-1*exp(logCumHaz[ss]))
	Br[ss] ~ dbin(cumFail[ss], Bn[ss])
	}

# Calculate HRs
	for (hh in 2:nTx) {
	hr[hh]<-exp(beta[hh])
	}

# Ranking plot
	for (ll in 1:nTx) {
		for (mm in 1:nTx) {
		rk[ll,mm]<- equals(ranked(beta[],mm),beta[ll])
		}
	}
}
```

Random effects analysis *(changes required to incorporate random effect in bold)*

```
model{

#Define Prior Distributions
	#on random tx effect variance
	sd~dunif(0,5)
	reTau <- 2/pow(sd,2)

	#On tx effect mean
	beta[1] <-0
	for (tt in 2:nTx){
	beta[tt]~dnorm(0,1.0E-6)
	}
```

```
		#On individual study baseline effect
	for(ss in 1:nStudies){
			alpha[ss] ~ dnorm(0,1.0E-6)
	}

#Define random effect
	for (ss in 1:nStudies){
		for(tt in 1:nTx){
		re[ss,tt]~dnorm(0,reTau)
		}
	}

#Fit data
	#For hazard ratio reporting studies
	for(ii in 1:LnObs ){
	Lmu[ii] <- alpha[Lstudy[ii]]*multi[ii] + re[Lstudy[ii],Ltx[ii]] -
	re[Lstudy[ii],Lbase[ii]] +  beta[Ltx[ii]] - beta[Lbase[ii]]
	Lprec[ii] <- 1/pow(Lse[ii],2)
	Lmean[ii] ~ dnorm(Lmu[ii],Lprec[ii])
	}

	#For binary data reporting studies
		 for(ss in 1:BnObs){
	logCumHaz[ss] <- alpha[Bstudy[ss]] + re[Bstudy[ss],Btx[ss]] -
	re[Bstudy[ss],Bbase[ss]] +  beta[Btx[ss]] - beta[Bbase[ss]]
	cumFail[ss] <- 1-exp(-1*exp(logCumHaz[ss]))
	Br[ss] ~ dbin(cumFail[ss], Bn[ss])
			}

# Calculate HRs
	for (hh in 2:nTx) {
	hr[hh]<-exp(beta[hh])
	}

# Ranking plot
	for (ll in 1:nTx) {
		for (mm in 1:nTx) {
		rk[ll,mm]<- equals(ranked(beta[],mm),beta[ll])
		}
	}
}
```
