# Supplementary material for: Mortality and drug therapy in patients with chronic obstructive pulmonary disease: a network meta-analysis
Source: BMC Pulm Med. 2015 Nov 11;15:145. doi: 10.1186/s12890-015-0138-4 (PMC4642642; doi:10.1186/s12890-015-0138-4)
Supplement: Additional file 2: Table S1. — Reasons for excluding full publications. Table 2. Risk of bias results. Table S3. Fixed effects network meta-analysis results. Hazard ratios compared to placebo (95% Credible Intervals). Table 4. Placebo mortality by treatment arm. (PDF 352 kb) [file 12890_2015_138_MOESM2_ESM.pdf]

Supplementary Tables

Appendix Table 1: Reasons for excluding full publications

Reference	Reason for exclusion
Aalbers R, Ayres J, Backer V, Decramer M, Lier PA, Magyar P, et al. Formoterol in patients with chronic obstructive pulmonary disease: a randomized, controlled, 3-month trial.[Erratum appears in Eur Respir J 2002 Jul;20(1):245]. European Respiratory Journal 2002;19(5):936-43.	<6 months
Ambrosino N, Foglio K, Balzano G, Paggiaro PL, Lessi P, Kesten S. Tiotropium and exercise training in COPD patients: Effects on dyspnea and exercise tolerance. International Journal of COPD 2008; 3 (4): 771-80.	mortality not reported
Anthonisen NR, Skeans MA, Wise RA, Manfreda J, Kanner RE, Connett JE, et al. The effects of a smoking cessation intervention on 14.5-year mortality: a randomized clinical trial.[Summary for patients in Ann Intern Med. 2005;142(4): 233-9	other
Auerbach D, Hill C, Baughman R, Boyars M, Braun S, Buist AS, et al. Routine nebulized ipratropium and albuterol together are better than either alone in COPD. Chest 112 (6) 1997: 1514-21.	<6 months
Bale G, Martinez-Camblor P, Burge PS, Soriano JB. Long-term mortality follow-up of the ISOLDE participants: causes of death during 13 years after trial completion. Respiratory Medicine 2008;102(10):1468-72.	Other
Barnes NC, Qiu YS, Pavord ID, Parker D, Davis PA, Zhu J, et al. Antiinflammatory effects of salmeterol/Fluticasone propionate in chronic obstructive lung disease. American Journal of Respiratory and Critical Care Medicine 2006;173(7):736-43.	<6 months
Barnes PJ, Pocock SJ, Magnussen H, Iqbal A, Kramer B, Higgins M, et al. Integrating indacaterol dose selection in a clinical study in COPD using an adaptive seamless design. Pulmonary Pharmacology and Therapeutics 2010;23(3):165-71.	<6 months
Baumgartner RA, Hanania NA, Calhoun WJ, Sahn SA, Sciarappa K, Hanrahan JP. Nebulized arformoterol in patients with COPD: a 12-week, multicenter, randomized, double-blind, double-dummy, placebo- and active-controlled trial. Clinical Therapeutics 2007;29(2):261-78.	<6 months
Bogdan MA, Aizawa H, Fukuchi Y, Mishima M, Nishimura M, Ichinose M. Efficacy and safety of inhaled formoterol 4.5 and 9 mug twice daily in Japanese and European COPD patients: Phase III study results. BMC Pulmonary Medicine 2011; 11	<6 months
Bone R, Boyars M, Braun SR, Buist AS, Campbell S, Chick T, et al. In chronic obstructive pulmonary disease, a combination of ipratropium and albuterol is more effective than either agent alone: An 85-day multicenter trial. Chest 1994; 105 (5):1411-9.	<6 months
Boorsma M, Lutter R, Van De Pol MA, Out TA, Jansen HM, Jonkers RE. Long-term effects of Budesonide on inflammatory status in COPD. COPD: Journal of Chronic Obstructive Pulmonary Disease 2008; 5 (2):97-104.	mortality not reported
Bourbeau J, Christodoulopoulos P, Maltais F, Yamauchi Y, Olivenstein R, Hamid Q. Effect of salmeterol/Fluticasone propionate on airway inflammation in COPD: a randomised controlled trial. Thorax 2007;62(11):938-43.	<6 months
Bourbeau J, Rouleau MY, Boucher S. Randomised controlled trial of inhaled corticosteroids in patients with chronic obstructive pulmonary disease. Thorax 53 (6) ()(pp 477-482), 1998 Date of Publication. 1998;(6):477-82.	<6 months
Bourbeau J. Inhaled corticosteroids and survival in chronic obstructive pulmonary disease. European Respiratory Journal 2003; 21 (2): 202-3.	review/letter/commentary
Boushey HA. Glucocorticoid therapy for chronic obstructive pulmonary disease. New England Journal of Medicine 1999; 340 (25):1990-1	review/letter/commentary
Boyd G, Morice AH, Pounsford JC, Siebert M, Peslis N, Crawford C. An evaluation of salmeterol in the treatment of chronic obstructive pulmonary disease (COPD).[Erratum appears in Eur Respir J 1997 Jul;10(7):1696]. European Respiratory Journal 1997;10(4):815-21.	<6 months

Reference	Reason for exclusion
Briggs DD, Jr., Covelli H, Lapidus R, Bhattycharya S, Kesten S, Cassino C. Improved daytime spirometric efficacy of Tiotropium compared with salmeterol in patients with COPD. Pulmonary Pharmacology & Therapeutics 2005;18(6):397-404.	<6 months
Brown SM. Tiotropium in combination with placebo, salmeterol, or Fluticasone salmeterol for chronic obstructive pulmonary disease: possible confounding effect of treatment withdrawal? Annals of Internal Medicine 2007;147(12):882-3.	review/letter/commentary
Buhl R, Dunn LJ, Disdier C, Lassen C, Amos C, Henley M, et al. Blinded 12-week comparison of once-daily indacaterol and Tiotropium in COPD. European Respiratory Journal 2011;38(4):797-803.	<6 months
Buist AS, Connett JE, Miller RD, Kanner RE, Owens GR, Voelker HT. Chronic Obstructive Pulmonary Disease Early Intervention Trial (Lung Health Study): Baseline characteristics of randomized participants. Chest 1993; 103 (6): 1863-72.	other
Buist AS. The US Lung Health Study. Respirology 1997; 2 (4): 303-7.	mortality not reported
Burge PS, Calverley PMA, Jones PW, Spencer S, Anderson JA. Prednisolone response in patients with chronic obstructive pulmonary disease: Results from the ISOLDE study. Thorax 2003; 58 (8):654-58.	mortality not reported
Calverley P, Pauwels DR, Lofdahl CG, Svensson K, Higenbottam T, Carlsson LG, et al. Relationship between respiratory symptoms and medical treatment in exacerbations of COPD.[Erratum appears in Eur Respir J. 2006 Feb;27(2):440]. European Respiratory Journal 2005 Sep;26(3):406-13.	mortality not reported
Calverley P, Pauwels R, Vestbo J, Jones P, Pride N, Gulsvik A, et al. Combined salmeterol and Fluticasone in the treatment of chronic obstructive pulmonary disease: a randomised controlled trial.[Erratum appears in Lancet. 2003 May 10;361(9369):1660]. Lancet 2003 Feb 8;361(9356):449-56	mortality not reported
Calverley PM, Spencer S, Willits L, Burge PS, Jones PW, IOSLDE Study Group. Withdrawal from treatment as an outcome in the ISOLDE study of COPD. Chest 2003;124(4):1350-6	other
Calverley PM, Stockley RA, Seemungal TA, Hagan G, Willits LR, Riley JH, et al. Reported pneumonia in patients with COPD: findings from the INSPIRE study. Chest 2011;139(3):505-12	other
Calverley PM, Rabe KF, Goehring U-M, Kristiansen S, Fabbri LM, Martinez FJ. Roflumilast in symptomatic chronic obstructive pulmonary disease: two randomised clinical trials. Lancet 2009; 374 (9691):685-94.	two trials combined
Casaburi R, Briggs DD, Jr., Donohue JF, Serby CW, Menjoge SS, Witek TJ, Jr. The spirometric efficacy of once-daily dosing with Tiotropium in stable COPD: A 13-week multicenter trial. Chest 2000;118 (5):1294-302	<6 months
Casaburi R, Mahler DA, Jones PW, Wanner A, San PG, ZuWallack RL, et al. A long-term evaluation of once-daily inhaled Tiotropium in chronic obstructive pulmonary disease. European Respiratory Journal 2002;19(2):217-24.	two trials combined
Cazzola M, AndÃ² F, Santus P, Ruggeri P, Marco F, Sanduzzi A, et al. A pilot study to assess the effects of combining Fluticasone propionate/salmeterol and Tiotropium on the airflow obstruction of patients with severe to very severe COPD. Pulmonary Pharmacology & Therapeutics 2007;20:556-61.	<6 months
Cazzola M, Califano C, Di PF, D'Amato M, Terzano C, Matera MG, et al. Acute effects of higher than customary doses of salmeterol and Salbutamol in patients with acute exacerbation of COPD. Respiratory Medicine 2002; ;96(10):790-5.	<6 months

Reference	Reason for exclusion
Cazzola M, Di LG, Di PF, Calderaro F, Testi R, Centanni S. Additive effects of salmeterol and Fluticasone or theophylline in COPD. Chest 2000;118(6):1576-81.	<6 months
Cazzola M, Noschese P, Centanni S, Santus P, Di MF, Spicuzza L, et al. Salmeterol/Fluticasone propionate in a Single Inhaler Device versus theophylline+Fluticasone propionate in patients with COPD. Pulmonary Pharmacology and Therapeutics 2004;17(3):141-5.	<6 months
Cazzola M, Pasqua F, Ferri L, Biscione G, Cardaci V, Matera MG. Rapid onset of bronchodilation with formoterol/Beclomethasone Modulite and formoterol/budesonide Turbuhaler as compared to formoterol alone in patients with COPD. Pulmonary Pharmacology and Therapeutics 2011 ;24(1):118-22.	<6 months
Celli B, Vestbo J, Jenkins CR, Jones PW, Ferguson GT, Calverley PM, et al. Sex differences in mortality and clinical expressions of patients with chronic obstructive pulmonary disease. The TORCH experience. American Journal of Respiratory & Critical Care Medicine 2011;183(3):317-22.	other
Charoenpan P, Kiatboonsri S, Uswanopakhun P, Vongvivat K, Sulaimanee P. The effects of inhaled Ipratropium bromide, fenoterol and their combination in COPD patients. Journal of the Medical Association of Thailand 1990; 73 (2): 91-95	<6 months
Chavannes NH, Schermer TR, Wouters EF, Akkermans RP, Dekhuijzen RP, Muris JW, et al. Predictive value and utility of oral steroid testing for treatment of COPD in primary care: the COOPT study. International Journal of COPD 2009; 4: 431-6.	mortality not reported
Colice GL. Nebulized bronchodilators for outpatient management of stable chronic obstructive pulmonary disease. The American Journal of Medicine 1996;100 (1 A):11S-8S.	<6 months
Conradson TB, Eklundh G, Olofsson B, Pahlm O, Persson G. Cardiac arrhythmias in patients with mild-to-moderate obstructive lung disease. Comparison of beta-agonist therapy alone and in combination with a xanthine derivative, enprofylline or theophylline. Chest 1985;88:537-42.	<6 months
Cook D, Guyatt G, Wong E, Goldstein R, Bedard M, Austin P, et al. Regular versus as-needed short-acting inhaled beta-agonist therapy for chronic obstructive pulmonary disease. American Journal of Respiratory & Critical Care Medicine 2001 ;163(1):85-90.	<6 months
Covelli H, Bhattacharya S, Cassino C, Conoscenti C, Kesten S. Absence of electrocardiographic findings and improved function with once-daily Tiotropium in patients with chronic obstructive pulmonary disease. Pharmacotherapy: The Journal of Human Pharmacology & Drug Therapy 2005 Dec;25(12):1708-18.	<6 months
Dahl R, Greefhorst LAPM, Nowak D, Nonikov V, Byrne AM, Thomson MH, et al. Inhaled formoterol dry powder versus ipratropium bromide in chronic obstructive pulmonary disease. American Journal of Respiratory and Critical Care Medicine 2001; 164 (5):778-84	<6 months
Dal Negro RW, Pomari C, Tognella S, Micheletto C. Salmeterol & Fluticasone 50 microg/250 microg bid in combination provides a better long-term control than salmeterol 50 microg bid alone and placebo in COPD patients already treated with theophylline. Pulmonary Pharmacology and Therapeutics 2003;16(4):241-6.	nonrandomised or unblinded
DalNegro R, Micheletto C, Trevison F, Tognells S, Pomari C, Spencer S. Salmeterol & Fluticasone 50µg/250µg bid vs Salmeterol 50µg bid and vs Placebo in the long• term treatment of COPD. American Journal of Respiratory and Critical Care Medicine 2002;165:A228.	review/letter/commentary
Dompeling E, van Schayck CP, Molema J, Folgering H, Van Grunsven PM, van WC. Inhaled Beclomethasone improves the course of asthma and COPD. European Respiratory Journal 1992;5(8):945-52.	other
Dompeling E, van Schayck CP, Van Grunsven PM, van Herwaarden CL, Akkermans R, Molema J, et al. Slowing the deterioration of asthma and chronic obstructive pulmonary disease observed during bronchodilator therapy by adding inhaled corticosteroids. A 4-year prospective study.	other

Reference	Reason for exclusion
Annals of Internal Medicine 1993;118(10):770-78	
Donohue JF, Menjoge S, Kesten S. Tolerance to bronchodilating effects of salmeterol in COPD. Respiratory Medicine 2003;97(9):1014-20.	mortality not reported
Dransfield MT, Cockcroft JR, Townsend RR, Coxson HO, Sharma SS, Rubin DB, et al. Effect of Fluticasone propionate/salmeterol on arterial stiffness in patients with COPD. Respiratory Medicine 2011;105(9):1322-30.	<6 months
Dusser D, Bravo ML, Iacono P. The effect of Tiotropium on exacerbations and airflow in patients with COPD.[Erratum appears in Eur Respir J. 2006 May;27(5):1076]. European Respiratory Journal 2006;27(3):547-55.	mortality not reported
Eichenhorn MS, Wise RA, Madhok TC, Gerald LB, Bailey WC, Tashkin DP, et al. Lack of long-term adverse adrenal effects from inhaled triamcinolone: Lung Health Study II. Chest 2003; 124 (1):p 57-62	mortality not reported
Fabbri LM, Calverley PM, Izquierdo-Alonso JL, Bundschuh DS, Brose M, Martinez FJ, et al. Roflumilast in moderate-to-severe chronic obstructive pulmonary disease treated with longacting bronchodilators: two randomised clinical trials. Lancet 2009;374(9691):695-703.	<6 months
Feldman G, Siler T, Prasad N, Jack D, Piggott S, Owen R, et al. Efficacy and safety of indacaterol 150 microg once-daily in COPD: a double-blind, randomised, 12-week study. BMC Pulmonary Medicine 2010;10:11.	<6 months
Fraser IM, Hyland RH, Hutcheon MA, MacKenzie RL, Ameli FM, Provan JL. Preliminary study of the effects of postoperative methylprednisolone therapy on lung function recovery in patients with chronic obstructive pulmonary disease. Clinical Pharmacy 1989; 8 (3):214-219	other
Freeman D, Lee A, Price D. Efficacy and safety of Tiotropium in COPD patients in primary care--the SPiRiva Usual CarE (SPRUCE) study. Respiratory Research 2007;8:45.	<6 months
Gaur SN, Rajpal S, Wasson S, Sanjay R, Sanjeev W. Therapeutic effect of inhaled Ipratropium bromide in patients of chronic obstructive pulmonary disease: a clinical trial. Indian Journal of Allergy and Applied Immunology 1997;11:67-71.	review/letter/commentary
Gimeno F, Veenen R. Placebo controlled comparison of the bronchodilator effects of Ipratropium bromide (Atrovent) inhaled as a dry powder and by metered dose inhaler in chronic obstructive pulmonary disease. Postgrad Med J 1987;20a.	review/letter/commentary
Gross NJ, Nelson HS, Lapidus RJ, Dunn L, Lynn L, Rinehart M, et al. Efficacy and safety of formoterol fumarate delivered by nebulization to COPD patients. Respiratory Medicine 2008;102:189-97.	<6 months
Hanania NA, Crater GD, Morris AN, Emmett AH, O'Dell DM, Niewoehner DE. Benefits of adding Fluticasone propionate/salmeterol to Tiotropium in moderate to severe COPD. Respiratory Medicine 2012; 106(1):91-101.	<6 months
Hattotuwa KL, Gizycki MJ, Ansari TW, Jeffery PK, Barnes NC. The effects of inhaled Fluticasone on airway inflammation in chronic obstructive pulmonary disease: a double-blind, placebo-controlled biopsy study. American Journal of Respiratory & Critical Care Medicine 2002;165(12):1592-6.	<6 months
Hoshino M, Ohtawa J. Effects of adding salmeterol/Fluticasone propionate to Tiotropium on airway dimensions in patients with chronic obstructive pulmonary disease. Respirology 2011; 16 (1): 95-101	<6 months
Johansson G, Lindberg A, Romberg K, Nordstrom L, Gerken F, Roquet A. Bronchodilator efficacy of Tiotropium in patients with mild to moderate COPD. Primary Care Respiratory Journal 2008;17(3):169-75.	<6 months
Johnell O, Pauwels R, Lofdahl CG, Laitinen LA, Postma DS, Pride NB, et al. Bone mineral density in patients with chronic obstructive pulmonary	mortality not reported

Reference	Reason for exclusion
disease treated with Budesonide Turbuhaler. European Respiratory Journal 2002;19(6):1058-63.	
Johnson SC, Gardener E, Hanley SP. A double blind double cross over comparison on high dose combined agonist and anticholinergic bronchodilators delivered by nebuliser or by inhaler and spacer in moderate to severe stable COPD patients. Thorax 2002;57:ii44	review/letter/commentary
Kamat SR, Hoskote VR, Store SD, Sanghavi B, Sheth UK. Comparison of oral Salbutamol and ephedrine in chronic obstructive pulmonary disease. Indian Journal of Chest Diseases & Allied Sciences 1978;20(2):54-62.	<6 months
Kaplan A. Effects of Tiotropium combined with either salmeterol or salmeterol/Fluticasone in moderate to severe COPD. Primary Care Respiratory Journal 2007;16 (4): 258-60.	review/letter/commentary
Kaptein AA, Brand PL, Dekker FW, Kerstjens HA, Postma DS, Sluiter HJ. Quality-of-life in a long-term multicentre trial in chronic nonspecific lung disease: assessment at baseline. The Dutch CNSLD Study Group. European Respiratory Journal 1993;6(10):1479-84.	other
Kinoshita M, Lee SH, Hang LW, Ichinose M, Hosoe M, Okino N, et al. Efficacy and safety of indacaterol 150 and 300 microg in chronic obstructive pulmonary disease patients from six Asian areas including Japan: a 12-week, placebo-controlled study. Respirology 2012;17(2):379-89.	<6 months
Korn S, Kerwin E, Atis S, Amos C, Owen R, Lassen C, et al. Indacaterol once-daily provides superior efficacy to salmeterol twice-daily in COPD: a 12-week study. Respiratory Medicine 2011;105(5):719-26.	<6 months
Kornmann O, Dahl R, Centanni S, Dogra A, Owen R, Lassen C, et al. Once-daily indacaterol versus twice-daily salmeterol for COPD: a placebo-controlled comparison. European Respiratory Journal 2011;37(2):273-9.	<6 months
Lacey RW, Khan GU, Martin GD. Theophylline in the management of chronic obstructive airways disease: a double blind comparison with amoxycillin and placebo. British Journal of Clinical Research 1995;6:151-61.	<6 months
Lapperre TS, Snoeck-Stroband JB, Gosman MM, Jansen DF, Van SA, Thiadens HA, et al. Effect of Fluticasone with and without salmeterol on pulmonary outcomes in chronic obstructive pulmonary disease: a randomized trial. Annals of Internal Medicine 2009;151(8):517-27.	mortality not reported
Lee SD, Hui DS, Mahayiddin AA, Roa CC, Jr., Kwa KH, Goehring UM, et al. Roflumilast in Asian patients with COPD: A randomized placebo-controlled trial. Respirology 2011; 16(8):1249-57.	<6 months
Mahler DA, D'Urzo A, Bateman ED, Ozkan SA, White T, Peckitt C, et al. Concurrent use of indacaterol plus Tiotropium in patients with COPD provides superior bronchodilation compared with Tiotropium alone: A randomised, double-blind comparison. Thorax 2012; 67 (9): 781-8.	two trials combined
Mahler DA, Donohue JF, Barbee RA, Goldman MD, Gross NJ, Wisniewski ME, et al. Efficacy of salmeterol xinafoate in the treatment of COPD. Chest 1999; 115 (4): 957-65.	<6 months
Mansori F, Nemat KA, Boskabady MH, Boskabady M. The effect of inhaled salmeterol, alone and in combination with Fluticasone propionate, on management of COPD patients. The Clinical Respiratory Journal 2010;4(4):241-7.	<6 months
Mirici A, Bektas Y, Ozbakis G, Erman Z. Effect of inhaled corticosteroids on respiratory function tests and airway inflammation in stable chronic obstructive pulmonary disease: A randomised, double-blind, placebo-controlled clinical trial. Clinical Drug Investigation 2001; 21 (12): 835-42.	<6 months
Moita J, Barbara C, Cardoso J, Costa R, Sousa M, Ruiz J, et al. Tiotropium improves FEV1 in patients with COPD irrespective of smoking status. Pulmonary pharmacology & therapeutics 2008; 21 (1): 146-51	<6 months

Reference	Reason for exclusion
Nava S, Karakurt S, Rampulla C, Braschi A, Fanfulla F. Salbutamol delivery during non-invasive mechanical ventilation in patients with chronic obstructive pulmonary disease: a randomized, controlled study. Intensive Care Medicine 2001;27(10):1627-35.	mortality not reported
Nelson HS, Gross NJ, Levine B, Kerwin EM, Rinehart M, Denis-Mize K, et al. Cardiac safety profile of nebulized formoterol in adults with COPD: a 12-week, multicenter, randomized, double- blind, double-dummy, placebo- and active-controlled trial.[Erratum appears in Clin Ther. 2009;31(4):920]. Clinical Therapeutics 2007t;29(10):2167-78.	<6 months
O'Donnell DE, Bredenbroker D, Brose M, Webb KA. Physiological effects of Roflumilast at rest and during exercise in COPD. European Respiratory Journal 2012;39(5):1104-12.	<6 months
Ozol D, Aysan T, Solak ZA, Mogulkoc N, Veral A, Sebik F. The effect of inhaled corticosteroids on bronchoalveolar lavage cells and IL-8 levels in stable COPD patients. Respiratory Medicine 2005; 99 (12): 1494-500.	mortality not reported
Paggiaro PL, Dahle R, Bakran I, Frith L, Hollingworth K, Efthimiou J. Multicentre randomised placebo-controlled trial of inhaled Fluticasone propionate in patients with chronic obstructive pulmonary disease. International COPD Study Group.[Erratum appears in Lancet 1998 Jun 27;351(9120):1968]. Lancet 1998;351(9105):773-80.	mortality not reported
Powrie DJ, Wilkinson TM, Donaldson GC, Jones P, Scrine K, Viel K, et al. Effect of Tiotropium on sputum and serum inflammatory markers and exacerbations in COPD. European Respiratory Journal 2007;30 (3): 472-8.	mortality not reported
Rabe KF, Bateman ED, O'Donnell D, Witte S, Bredenbroker D, Bethke TD. Roflumilast--an oral anti-inflammatory treatment for chronic obstructive pulmonary disease: a randomised controlled trial. Lancet 2005;366(9485):563-71.	mortality not reported
Rand CS, Nides M, Cowles MK, Wise RA, Connett J. Long-term metered-dose inhaler adherence in a clinical trial. The Lung Health Study Research Group. American Journal of Respiratory and Critical Care Medicine 1995;152(2):580-8.	other
Reid DW, Wen Y, Johns DP, Williams TJ, Ward C, Walters EH. Bronchodilator reversibility, airway eosinophilia and anti-inflammatory effects of inhaled Fluticasone in COPD are not related. Respirology 2008;13(6):799-809.	mortality not reported
Rennard SI, Anderson W, ZuWallack R, Broughton J, Bailey W, Friedman M, et al. Use of a long-acting inhaled beta2-adrenergic agonist, salmeterol xinafoate, in patients with chronic obstructive pulmonary disease. American Journal of Respiratory and Critical Care Medicine 2001; 163 (5):1087-92.	<6 months
Rutten-Van MM, Roos B, Van Noord JA. An empirical comparison of the St George's Respiratory Questionnaire (SGRQ) and the Chronic Respiratory Disease Questionnaire (CRQ) in a clinical trial setting. Thorax 1999; 54 (11):995-1003.	<6 months
Scanlon PD, Connett JE, Wise RA, Tashkin DP, Madhok T, Skeans M, et al. Loss of bone density with inhaled triamcinolone in Lung Health Study II. American Journal of Respiratory and Critical Care Medicine 2004;170(12):1302-9.	other
Schermer TR, Albers JM, Verblackt HW, Costongs RJ, Westers P. Lower inhaled steroid requirement with a Fluticasone/salmeterol combination in family practice patients with asthma or COPD. Family Practice 2007;24(2):181-8.	<6 months
Senderovitz T, Vestbo J, Frandsen J, Maltbaek N, Norgaard M, Nielsen C, et al. Steroid reversibility test followed by inhaled Budesonide or placebo in outpatients with stable chronic obstructive pulmonary disease. Respiratory Medicine 1999;93:715-8.	mortality not reported
Shaker SB, Stavngaard T, Laursen LC, Stoel BC, Dirksen A. Rapid fall in lung density following smoking cessation in COPD. COPD: Journal of COPD 2011; 8 (1):2-7	mortality not reported

Reference	Reason for exclusion
Sharafkhaneh A, Southard JG, Goldman M, Uryniak T, Martin UJ. Effect of Budesonide/formoterol pMDI on COPD exacerbations: A double-blind, randomized study. COPD: Journal of Chronic Obstructive Pulmonary Disease 2012; 9 (1):85	review/letter/commentary
Taccola M, Bancalari L, Ghignoni G, Paggiaro PL. Salmeterol versus slow-release theophylline in patients with reversible obstructive pulmonary disease. Monaldi archives for chest disease = Archivio Monaldi per le malattie del torace / Fondazione clinica del lavoro , IRCCS [and] Istituto di clinica tisiologica e malattie apparato respiratorio , Universite di Napoli, Secondo ateneo 1999;54:302-6.	nonrandomised or unblinded
Tashkin DP, Celli B, Kesten S, Lystig T, Mehra S, Decramer M. Long-term efficacy of Tiotropium in relation to smoking status in the UPLIFT trial.[Erratum appears in Eur Respir J. 2010 May;35(5):1195]. European Respiratory Journal 2010;35(2):287-94.	other
Taylor J, Kotch A, Rice K, Ghafouri M, Kurland CL, Fagan NM, et al. Ipratropium bromide hydrofluoroalkane inhalation aerosol is safe and effective in patients with COPD. Chest 2001;120(4):1253-61.	<6 months
Tsagaraki V, Amfilochiou A, Markantonis SL. Evidence of tachyphylaxis associated with salmeterol treatment of chronic obstructive pulmonary disease patients. International Journal of Clinical Practice 2006;60:415-21	<6 months
Tzani P, Crisafulli E, Nicolini G, Aiello M, Chetta A, Clini EM, et al. Effects of Beclomethasone/formoterol fixed combination on lung hyperinflation and dyspnea in COPD patients. International Journal of COPD 2011; 6 (1):503-9.	<6 months
van Grunsven P, Schermer T, Akkermans R, Albers M, van den Boom G, van SO, et al. Short- and long-term efficacy of Fluticasone propionate in subjects with early signs and symptoms of chronic obstructive pulmonary disease. Results of the DIMCA study. Respiratory Medicine 2003; 97 (12):1303-12.	<6 months
Van Grunsven PM, van Schayck CP, Van DM, Van Herwaarden CLA, Akkermans RP, van WC. Compliance during long-term treatment with Fluticasone propionate in subjects with early signs of asthma or chronic obstructive pulmonary disease (COPD): Results of the detection, intervention, and monitoring ram of COPD and asthma (DIMCA) study. Journal of Asthma 2000; 37 (3):225-34.	mortality not reported
Van Noord JA, Bantje T, Eland ME, Korducki L, Cornelissen PJG. A randomised controlled comparison of Tiotropium and ipratropium in the treatment of chronic obstructive pulmonary disease. Thorax 2000; 55 (4): 289-94.	<6 months
Van Noord JA, de Munck DR, Bantje TA, Hop WC, Akveld ML, Bommer AM. Long-term treatment of chronic obstructive pulmonary disease with salmeterol and the additive effect of ipratropium. European Respiratory Journal 2000;15(5):878-85.	<6 months
Verkindre C, Bart F, Aguilaniu B, Fortin F, Guerin J-C, Le MC, et al. The effect of Tiotropium on hyperinflation and exercise capacity in chronic obstructive pulmonary disease. Respiration 2006; 73 (4):420-7.	<6 months
Vincken W, Van Noord JA, Greefhorst AP, Bantje TA, Kesten S, Korducki L, et al. Improved health outcomes in patients with COPD during 1 yr's treatment with tiotropium. European Respiratory Journal 2002;19(2):209-16.	two trials combined
Voduc N, Alvarez GG, Amjadi K, Tessier C, Sabri E, Aaron SD. Effect of theophylline on exercise capacity in COPD patients treated with combination long-acting bronchodilator therapy: A pilot study. International Journal of COPD 2012; 7:245-52.	<6 months
Voshaar T, Lapidus R, Maleki-Yazdi R, Timmer W, Rubin E, Lowe L, et al. A randomized study of Tiotropium Respimat Soft MistTM Inhaler vs. ipratropium pMDI in COPD. Respiratory Medicine 2008; 102 (1):32-41.	<6 months
Wadbo M, Lofdahl CG, Larsson K, Skoogh BE, Tornling G, Arwestrom E, et al. Effects of formoterol and ipratropium bromide in COPD: a 3-month placebo-controlled study. European Respiratory Journal 2002;20(5):1138-46.	<6 months

Reference	Reason for exclusion
Weir DC, Bale GA, Bright P, Sherwood BP. A double-blind placebo-controlled study of the effect of inhaled Beclomethasone dipropionate for 2 years in patients with nonasthmatic chronic obstructive pulmonary disease. Clinical and Experimental Allergy 1999;29:125-28	mortality not reported
Welte T, Miravitlles M, Hernandez P, Eriksson G, Peterson S, Polanowski T, et al. Efficacy and tolerability of Budesonide/formoterol added to Tiotropium in patients with chronic obstructive pulmonary disease. American Journal of Respiratory and Critical Care Medicine 2009; 180 (8):741-50.	<6 months
Whitford H, Walters EH, Levvey B, Kotsimbos T, Orsida B, Ward C, et al. Addition of inhaled corticosteroids to systemic immunosuppression after lung transplantation: a doubleâ€• blind, placebo-controlled trial. Transplantation 2002;73:1793-9.	<6 months
Yildirim E, Yildiz F, Kacar OS, Basyigit I, Boyaci H, Ilgazli A. Effects of Different Combined Bronchodilator Therapies on Airway Inflammation in COPD. Clinical Drug Investigation 2005;25(7):453-61.	<6 months
Yildiz F, Basyigit I, Yildirim E, Boyaci H, Ilgazli A. Different bronchodilator combinations have similar effects on health status in COPD. Pulmonary Pharmacology and Therapeutics 2006;19(2):101-6.	<6 months
Zhou Y, Wang X, Zeng X, Qiu R, Xie J, Liu S, et al. Positive benefits of theophylline in a randomized, double-blind, parallel-group, placebo-controlled study of low-dose, slow-release theophylline in the treatment of COPD for 1 year. Respirology 2006;11(5):603-10.	mortality not reported
ZuWallack RL, Mahler DA, Reilly D, Church N, Emmett A, Rickard K, et al. Salmeterol plus theophylline combination therapy in the treatment of COPD. Chest 2001; 119 (6): 1661-70	<6 months

Appendix Table 2 Risk of bias results

Trial	Method of randomisation	Allocation concealment	Blinding of participants/ personnel	Blinding of outcome assessment	Incomplete outcome data	Selective reporting
Aaron 2007	+	+	+	+	+	+
Anzueto 2009	+	+	+	+	?	+
Bateman 2010	+	+	+	+	+	+
ISOLDE	+	+	+	+	+	+
Calverley 2003	?	+	+	+	+	+
TORCH	+	+	+	+	+	+
Calverley 2007	+	+	+	+	+	+
M2-124	+	+	+	+	+	+
M2-125	+	+	+	+	+	+
Calverley 2010	+	+	+	+	+	+
Campbell 2005	+	+	+	+	+	+
Casaburi 2005	?	+	+	+	+	+
Chan 2007	?	+	+	+	?	+
Choudhury 2007	+	+	+	+	+	+
INVOLVE	?	+	+	+	+	+
Donohue 2002	?	+	+	+	?	+
INHANCE	?	+	?	+	?	+
Ferguson 2008	?	+	+	+	+	+
Hanania 2003	?	+	+	+	?	+
VIVACE	?	+	+	+	+	+
Kerstjens 1992	+	+	+	+	?	?
INLIGHT-2	+	+	+	+	?	+
Mahler 2002	?	+	+	+	?	+
Niewoehner 2005	+	+	+	+	+	+
EUROSCOP	?	+	+	+	?	+
Rennard 2009	?	+	+	+	+	+
FICOPD II	?	+	+	+	+	+
Schermer 2009	+	+	+	+	+	+
Shaker 2009	+	+	+	+	?	+
Stockley 2006	+	+	+	+	?	+
Szafranski 2003	?	?	?	+	?	+
Tashkin 2008	+	+	+	+	+	+
UPLIFT	+	+	+	+	+	+
Tonnel 2008	+	+	+	+	?	+
COPE	+	+	+	+	?	+
CCLS	+	+	+	+	?	+
Vogelmeier 2008	?	+	+	+	+	+
POET-COPD	+	+	+	+	?	+
INSPIRE	+	+	+	+	+	+
LHS	?	+	+	+	?	+
Zheng 2007	?	?	?	+	-	+
Zhong 2012	+	+	+	+	-	+

[+] low risk of bias; [?] unclear risk of bias; [-] high risk of bias
Sensitivity analysis (G) excluded studies where two or more criteria were assessed as unclear or high risk

Appendix Table 3: Fixed effects network meta-analysis results. Hazard ratios compared to placebo (95% Credible Intervals).

		(A)	(B)	(C)	(D)	(E)	(G)	(H)	(I)
**Treatment**	**Base case**	**Death OT (40)†**	**Adjusting for baseline FEV_1 % predicted (40)**	**Baseline FEV_1 <65% (36)**	**Licensed doses (29)‡**	**≥48 wk (27)**	**Quality criteria (31)**	**Additional studies from Dong et al (53)**	**Tiotropium by inhaler type (TIOSPIR) (41)**
TIO [H]	0.93 (0.83, 1.04)	0.90 (0.81, 1.02)	0.93 (0.83, 1.04)	0.93 (0.83, 1.04)	0.92 (0.81, 1.03)	0.93 (0.83, 1.05)	0.92 (0.82, 1.04)	0.93 (0.83, 1.04)	0.93 (0.83, 1.04)
TIO [S]	-	-	-	-	-	-	-	-	0.93 (0.80, 1.07)
TIO + SAL	1.58 (0.42, 6.49)	1.50 (0.41, 6.00)	1.54 (0.42, 6.10)	1.53 (0.42, 6.25)	-	1.55 (0.42, 6.12)	1.55 (0.42, 6.22)	1.56 (0.42, 6.46)	1.54 (0.42, 6.21)
TIO + SFC	1.61 (0.43, 6.62)	1.54 (0.42, 6.13)	1.57 (0.43, 6.25)	1.56 (0.42, 6.35)	-	1.58 (0.43, 6.33)	1.59 (0.43, 6.39)	1.59 (0.43, 6.51)	1.57 (0.42, 6.37)
SAL	0.90 (0.76, 1.05)	0.88 (0.75, 1.03)	0.89 (0.76, 1.06)	0.89 (0.76, 1.05)	0.56 (0.28, 1.11)	0.91 (0.77, 1.07)	0.90 (0.76, 1.06)	0.90 (0.77, 1.06)	0.90 (0.76, 1.06)
SFC	0.79 (0.67, 0.94)	0.78 (0.66, 0.93)	0.79 (0.66, 0.94)	0.79 (0.66, 0.94)	0.56 (0.35, 0.88)	0.80 (0.67, 0.95)	0.80 (0.67, 0.95)	0.78 (0.66, 0.93)	0.79 (0.67, 0.94)
FP	1.06 (0.90, 1.25)	1.04 (0.89, 1.22)	1.05 (0.90, 1.24)	1.04 (0.89, 1.22)	-	1.07 (0.91, 1.25)	1.06 (0.91, 1.25)	1.04 (0.89, 1.22)	1.06 (0.90, 1.24)
FORM	1.14 (0.70, 1.84)	1.13 (0.66, 1.93)	1.14 (0.70, 1.84)	1.15 (0.70, 1.89)	0.93 (0.79, 1.1)	1.14 (0.70, 1.87)	1.33 (0.76, 2.33)	1.14 (0.70, 1.85)	1.14 (0.71, 1.84)
BUDE	0.84 (0.52, 1.35)	0.85 (0.52, 1.36)	0.86 (0.52, 1.41)	0.87 (0.47, 1.57)	-	0.82 (0.50, 1.32)	0.93 (0.49, 1.75)	0.84 (0.52, 1.35)	0.84 (0.52, 1.35)
BUDE + FORM	1.13 (0.69, 1.86)	1.17 (0.68, 2.00)	1.12 (0.68, 1.84)	1.14 (0.69, 1.89)	0.85 (0.71, 1.03)	1.03 (0.61, 1.73)	1.34 (0.75, 2.39)	1.13 (0.70, 1.84)	1.13 (0.69, 1.83)
ROFL	0.88 (0.61, 1.26)	0.87 (0.61, 1.26)	0.86 (0.57, 1.29)	0.88 (0.61, 1.26)	0.88 (0.60, 1.27)	0.88 (0.61, 1.26)	0.88 (0.61, 1.26)	0.88 (0.61, 1.26)	0.87 (0.61, 1.26)
BECL + FORM	1.01 (0.13, 6.30)	1.03 (0.13, 6.28)	1.00 (0.12, 6.18)	1.01 (0.12, 6.25)	0.78 (0.10, 4.56)	0.97 (0.12, 5.99)	1.19 (0.14, 7.39)	1.01 (0.13, 6.12)	1.01 (0.13, 6.20)
INDA	0.28 (0.08, 0.84)	0.26 (0.06, 0.91)	0.29 (0.08, 0.86)	0.29 (0.08, 0.85)	0.28 (0.06, 0.93)	0.17 (0.03, 0.78)	0.26 (0.06, 0.92)	0.29 (0.08, 0.85)	0.29 (0.08, 0.85)
TRIA	0.78 (0.39, 1.54)	0.78 (0.39, 1.54)	0.81 (0.37, 1.80)	-	-	0.78 (0.39, 1.54)	-	0.78 (0.39, 1.54)	0.78 (0.39, 1.54)
bCov	-	-	*1.00 (0.98, 1.02)*	-	-	-	-	-	-

Number in title row indicates number of studies included in each sensitivity analysis; results are presented as Hazard Ratios compared to placebo (HR (95% CrI));

TIO– Tiotropium; SAL – Salmeterol; SFC – Salmeterol + Fluticasone propionate; FP - Fluticasone propionate; FORM – Formoterol; BUDE – Budesonide; ROFL – Roflumilast; BECL – Beclomethasone; INDA – Indacaterol; TRIA – Triamcinolone;

[H] – Handihaler, [S] –SoftMist

† – six studies reported on treatment death: Chan (2007), Donohue (2010), Rennard (2009), Vogelmeier (2011), Tashkin (2008)

‡ – 11 studies were excluded for including patients treated with unlicensed doses: Pauwels (1999), Shaker (2009), Wise (2000), Calverley (2003), Van Valk (2002), Vestbo (1999), Zhong (2012), Schermer (2009), Aaron (2007), Burge (2000), Choudhury (2007); . Only the following interventions and doses were considered: beclomethasone + formoterol (200/20 µg bid), budesonide + formoterol (320/9 µg and 400/12 µg bid), formoterol (12 µg and 24 µg bid), indacaterol (150 µg and 300 µg od), roflumilast (500 µg od), salmeterol (50 µg bid), SFC (50/250 µg and 50/500 µg bid), theophylline (200 mg and 300mg bid), tiotropium (5 µg and 18 µg od) and terbutaline + placebo (2000 µg/NA bid).

Subgroup analysis F including only studies powered to detect mortality was not conducted

Appendix Table 4: Placebo mortality by treatment arm

Treatment	PLACEBO ARMS		
	Deaths - ITT	Person years	Deaths/PY *1000
FP	274	6331	43.3
TIO + SFC	NA	NA	-
TIO + SAL	NA	NA	-
TIO	567	15224	37.2
SFC	234	4809	48.7
SAL	246	5231	47.0
ROFL	62	2307	26.9
FORM + TERA	NA	NA	-
BUDE + FORM	23	1092	21.1
FORM	29	1840	15.8
BECL + FORM	NA	NA	-
TRIA	19	1671	11.4
BUDE	35	3483	10.0
INDA	8	809	9.9
TERA	NA	NA	-
Total	1498	43114	34.7

TIO– Tiotropium; SAL – Salmeterol; SFC – Salmeterol + Fluticasone propionate; FP - Fluticasone propionate; FORM – Formoterol; BUDE – Budesonide; ROFL – Roflumilast; BECL – Beclomethasone; INDA – Indacaterol; TRIA – Triamcinolone
